# Effect of Sodium Bicarbonate on Systolic Blood Pressure in CKD: A Systematic Review and Meta-Analysis

**DOI:** 10.2215/CJN.0000000000000119

**Published:** 2023-03-09

**Authors:** Beverley Beynon-Cobb, Panayiotis Louca, Ewout J. Hoorn, Cristina Menni, Sandosh Padmanabhan

**Affiliations:** 1 Department of Nutrition & Dietetics, University Hospitals Coventry & Warwickshire NHS Trust, Coventry, United Kingdom; 2 Department of Twin Research and Genetic Epidemiology, King's College London, London, United Kingdom; 3 Department of Internal Medicine, Divisions of Nephrology and Transplantation, Erasmus Medical Center, University Medical Center Rotterdam, Rotterdam, The Netherlands; 4 BHF Glasgow Cardiovascular Research Centre, School of Cardiovascular & Metabolic Health, University of Glasgow, Glasgow, United Kingdom

**Keywords:** chronic metabolic acidosis, sodium bicarbonate, systolic blood pressure

## Abstract

**Background:**

Individuals with CKD are at a higher risk of cardiovascular morbidity and mortality. Acidosis is positively correlated with CKD progression and elevated systolic BP. Sodium bicarbonate is an efficacious treatment of acidosis, although this may also increase systolic BP. In this systematic review and meta-analysis, we summarize the evidence evaluating systolic BP and antihypertensive medication change (which may indicate systolic BP change) in response to sodium bicarbonate therapy in individuals with CKD.

**Methods:**

Medical Literature Analysis and Retrieval System Online, Excerpta Medica database, Cumulative Index to Nursing and Allied Health Literature, Allied and Complementary Medicine Database, Cochrane Central Register of Controlled Trials, and World Health Organization (WHO) trials registry databases were searched for randomized control trials where sodium bicarbonate was compared with placebo/usual care in CKD stage G1–5 non–dialysis-dependent populations. Random effects meta-analyses were used to evaluate changes in systolic BP and BP-modifying drugs after sodium bicarbonate intervention.

**Results:**

Fourteen randomized control trials (2110 individuals, median follow-up 27 [interquartile range 97] weeks, mean age 60 [SD 10] years, mean systolic BP 136 [SD 17] mm Hg, mean eGFR 38 [SD 10] ml/min, mean serum bicarbonate 22 [SD 4] mmol/L) were eligible for inclusion. Meta-analysis suggested that sodium bicarbonate did not influence systolic BP in individuals with CKD stage G1–5. Results were consistent when stratifying by dose of sodium bicarbonate or duration of intervention. Similarly, there was no significant increase in the use of antihypertensive medication or diuretics in individuals taking sodium bicarbonate, whereas there was a greater decrease in antihypertensive medication use in individuals taking sodium bicarbonate compared with controls.

**Conclusions:**

Our results suggest, with moderate certainty, that sodium bicarbonate supplementation does not adversely affect systolic BP in CKD or negatively influence antihypertensive medication requirements.

## Visual Abstract

**Figure:**
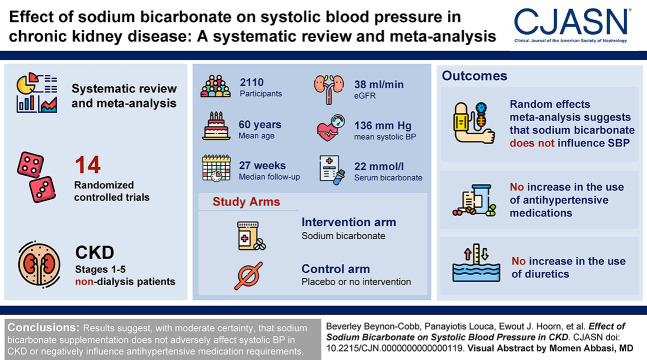

## Introduction

Hypertension is a modifiable risk factor in the development of CKD and associated cardiovascular disease.^1^ As kidney function declines, systolic BP progressively increases.^2^ The Chronic Renal Insufficiency Cohort study suggests that adults with stage G3–5 CKD are up to three times more likely to suffer from hypertension compared with the general population.^2^ Kidney Disease Improving Global Outcomes (KDIGO) guidance focuses on the treatment of systolic BP to manage cardiovascular risk in CKD and recommends systolic BP treatment targets of <120 mm Hg for nondiabetic individuals with high BP and CKD.^3^ Clinical strategies to achieve these BP targets include dietary sodium chloride (salt) restriction.

Metabolic acidosis (serum bicarbonate level<22 mmol/L) is associated with poorer health outcomes.^4^ Metabolic acidosis results from an inability to maintain acid-base balance by excreting organic acids and hydrogen ions while conserving bicarbonate ions. This increases dependency on generating bicarbonate ions by ammoniagenesis, an inflammatory process that has been linked with deterioration of kidney function.^5^ The prevalence of metabolic acidosis varies from 7% in CKD stage G2 to 37% in CKD stage G4.^6^ For acidotic individuals with CKD, KDIGO guidance^7^ recommends supplementation with sodium bicarbonate, unless there is a clinical concern that the associated sodium load will exacerbate hypertension and/or fluid overload. A large body of evidence supports a direct relationship between sodium consumption and hypertension, which is exacerbated in individuals with CKD.^8^ This relationship has raised clinical concerns regarding the use of sodium bicarbonate therapy in CKD. However, research suggests that sodium salts have varying effects on BP depending on their anion base. Indeed, some studies suggest that sodium chloride has a detrimental effect on BP, whereas sodium bicarbonate may improve BP.^9^ Large trials supporting these findings are lacking, and recent meta-analyses did not analyze changes in antihypertensive medication or diuretics, which may mask BP changes.^10–12^ In this study, we aimed to conduct a systematic review and meta-analysis of randomized control trials (RCTs) to evaluate the effect of sodium bicarbonate therapy (or its precursor sodium citrate) on systolic BP in CKD stage G1–5 non–dialysis-dependent population and analyze antihypertensive and diuretic medication changes.

## Methods

This systematic review was conducted using a prepublished protocol produced using Preferred Reporting Items for Systematic review and Meta‐Analysis Protocols guidance and registered with the International Prospective Register of Systematic Reviews (prospective register of systematic reviews in health and social care Centre for Reviews and Dissemination 58933), available at https://www.crd.york.ac.uk/PROSPERO/display_record.php?RecordID=58933. This systematic review is an update to an unpublished systematic review undertaken for a master's degree by one author (B.BC) available at https://pureportal.coventry.ac.uk/en/studentTheses/the-effect-of-sodium-bicarbonate-on-blood-pressure-in-chronic-kid.

### Search Strategy and Study Selection

The following electronic databases were searched: Medical Literature Analysis and Retrieval System Online, Excerpta Medica database, Cumulative Index to Nursing and Allied Health Literature, Allied and Complementary Medicine Database, Cochrane Central Register of Controlled Trials, and the WHO trials registry database for articles published till January 2021.

#### RCT Inclusion Criteria

The inclusion criteria for RCT were: non-dialysis human participants with CKD stage G1–5, an intervention arm providing sodium bicarbonate or sodium citrate, a control arm providing a placebo or no intervention, reported baseline and end-of-intervention systolic BP, and reported change in antihypertensive and diuretic medications.

#### RCT Exclusion Criteria

The exclusion criteria were as follows: participants undergoing KRT of any form, including transplantation; people with AKI; or lack of a control arm.

No restrictions were applied for the presence of clinical acidosis, language, date of publication, or study duration. See Supplemental Methods for further details on the search strings.

If multiple publications of the same trials were identified, outdated publications were discarded and the publication containing the most complete or most recently updated dataset was included. Missing or unpublished data were requested from the relevant study investigators; studies where data were refused or no contact was possible were excluded.

### Data Extraction

Outcome data were extracted independently by two authors. Differences identified were discussed and agreed to finalize data documentation. Extracted data included patient demographic details, study outcomes (baseline and end-of-study values for systolic BP, serum bicarbonate, antihypertensive and diuretic medications), dose of sodium bicarbonate, duration of the study, and adverse events. Extracted data were uploaded to RevMan5.^13^

### Outcomes Assessed

The primary outcomes assessed were change in mean systolic BP and antihypertensive and diuretic medications from baseline to end of intervention. Secondary outcomes included changes in mean serum bicarbonate and the intervention dose of sodium bicarbonate.

### Statistical Analyses

For continuous data outcomes (systolic BP and mean serum bicarbonate), the effect measure is expressed as a mean difference and calculated using a Der Simonian–Laird random-effects model. For these outcomes, the average systolic BP and serum bicarbonate at the end of the intervention period were extracted with SD, where available. Where SD values were not expressed, they were imputed from the data available as per Cochrane guidelines. The use of end-of-intervention data represented a deviation from the review protocol, which stated that mean difference values would be used. Unfortunately, mean difference values were not available for most of the studies. The imputation of mean difference values as per the Cochrane Handbook was not possible because a correlation coefficient could not be imputed and applied from other included studies because of the heterogeneity of those studies. For dichotomous outcomes relating to change in antihypertensive and diuretic medications, the effect measure is expressed as a risk ratio (RR) with 95% confidence intervals (CIs) calculated using a Mantel–Haenszel random-effects model.

Subgroup analyses were conducted for stage of CKD, dose of sodium bicarbonate, and duration of the study. One study^14^ included a control and two intervention arms using different doses of sodium bicarbonate. To avoid double counting of the control data, two meta-analyses were conducted for each outcome, one including the high-dose and the other the low-dose sodium bicarbonate intervention group. We stratified the dose of sodium bicarbonate as follows: low dose <2.5 mEqM/kg, medium dose 0.26–0.5 mEqm/kg, and high dose >0.51 mEqM/kg. In a 70-kg individual, this would equate to sodium bicarbonate doses of <1.2, 1.8–2.9, and >3 g for low-dose, medium-dose, and high-dose groups, respectively, which reflects local practice. For the duration of intervention, we classified the studies as follows: short term 4–12 weeks, medium term 24–28 weeks, and long term 104–520 weeks.

Heterogeneity across the studies was estimated using χ^2^ (Cochrane Q) and I^2^ statistics. A χ^2^ <0.05 suggests the presence of heterogeneity while I^2^ values of 25%, 50%, and 75% are used to quantify heterogeneity as low, medium, and high, respectively.

### Risk of Bias

The methodological quality of each included study was assessed using the Cochrane Risk of Bias 2.0 assessment tool^15^ and documented in RevMan5.^13^ Internal validity (bias) of each study was defined by five domains: *(1)* the randomization process, *(2)* deviations from intended interventions, *(3)* missing outcome data, *(4)* measurement of the outcome, and *(5)* the selection of the reported result. These domains are then used to form an overall risk of bias, categorized as low, medium, or high.

Sources of clinical heterogeneity were explored using a sensitivity analysis, excluding studies with a high risk of bias; where systolic BP was not a specified primary or secondary outcome; where a placebo was not used; where dose of sodium bicarbonate was based on bodyweight of participants or absolute dosing; and where the mean serum bicarbonate was “normal” (*i.e.*, >22 mmol/L) at baseline. The potential for small study effects (publication bias) was assessed by testing funnel plot asymmetry, using Stata (Version 17). To evaluate heterogeneity in treatment effects between studies, data were exported from RevMan5 to Stata17 for meta-regression analysis.

## Results

### Search Results and Description of Included Studies

**Figure 1:**
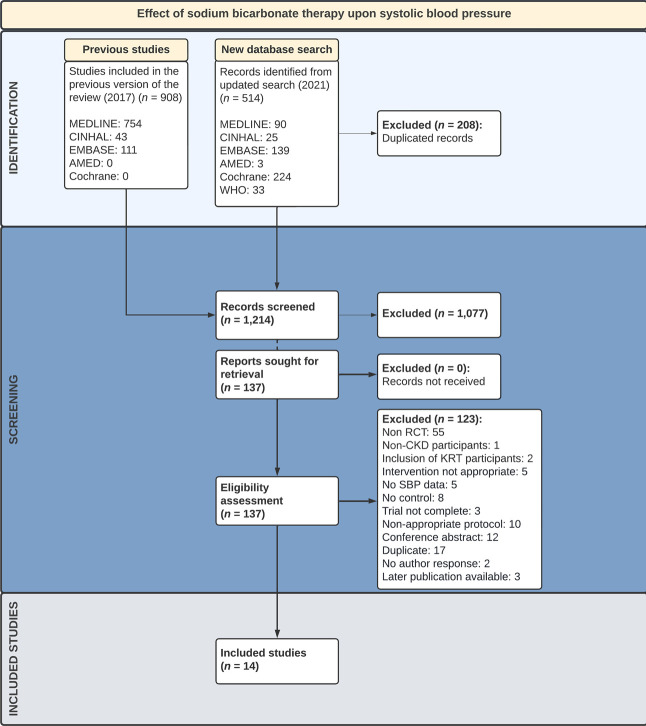
**Preferred Reporting Items for Systematic review and Meta‐Analysis Protocols flow diagram.** AMED, Allied and Complementary Medicine Database; CINHAL, Cumulative Index to Nursing and Allied Health Literature; Cochrane, Cochrane Central Register of Controlled Trials; EMBASE, Excerpta Medica database; MEDLINE, Medical Literature Analysis and Retrieval System Online; RCT, randomized control trial; SBP, systolic BP; WHO, World Health Organization.

The searches were conducted as per the search criteria and completed in January 2021. A total of 1389 publications were identified and screened (Figure 1).

### Included Studies

Fourteen studies, including 2110 participants (median 73, range 30–740, interquartile range [IQR] 135), were included. Descriptive characteristics of the included studies are listed in Table 1. Briefly, the median follow-up time was 27 weeks (range 4–520, IQR 97); the median age of participants was 60 years (range: 41–74, IQR 15); and 62% of the population were male. Six trials used a placebo while eight trials used standard care for the control group. All sodium bicarbonate supplements were administered orally, and the median dose was 0.5 mEqM/kg (range 0.2–1.2, IQR 0.2). For four studies,^16–19^ absolute doses were administered. Mean dose and mean weight of the sample were used to calculate a weight-based dosing to standardize the dosing between studies. The mean eGFR was 39 (SD 10) ml/min. While most of the studies included participants with stage G4–G5 CKD, two trials included participants with stage G1 or G2 CKD. The mean systolic BP and mean serum bicarbonate values at trial entry were 136 (SD 16) mm Hg and 22 (SD 4) mmol/L, respectively. Metabolic acidosis at entry was not an inclusion criterion for all trials because the primary outcomes for these trials were other biomarkers, such as urinary ammonia, or functional capacity measures (*e.g.*, sit-to-stand tests). Consequently, only seven trials had mean serum bicarbonate values of <22 mmol/L at entry. Two trials included three arms: a sodium bicarbonate intervention, a control, and an alkaline diet or sodium chloride arm; data that did not pertain to this review were not included.

**Table 1:** Characteristics of included studies

Study	*n*	Age (yr)	Male (%)	G-Stage CKD	Study Duration (wk)	NaHCO_3_ Dose mEq/kg	P or SC	Serum HCO_3_ (mmol/L)	Systolic BP (mm Hg)
Bovee 2020^16^	30	62±15	78	4	4	0.5	SC	21.7±3.3	137±16
de-Brito 2009^22^	129	58±2.5	52	4	104	0.2	SC	19.9±15.4	124±10
Di Iorio 2019^27^	740	68±14.9	62	3	206	0.6	SC	21.5±2.4	128±18
Dubey 2020^17^	188	50±11.5	71	4	26	0.5	SC	18±2.3	126±21
Goraya 2012^20^	53	50±8.9	47	1	4	0.5	SC	26.4±0.8	133±6
Goraya 2019 (CKD 2)^23^	80	51±8.4	48	2	520	0.5	SC	26±0.71	155±14
Goraya 2019 (CKD 3)^23^	66	54±5.1	44	3	260	0.3	SC	23±0.6	162±11
Jones 2019^18^	39	69±10.6	77	4	4	0.2	P	23.7±2.9	140±18
Kendrick 2018^24^	40	59±12	50	4	6	0.4	SC	19.5±2.3	132±20
Mathur 2006^29^	40	41±14.1	60	4	12	1.2	P	19.4±4.7	134±7
Melamed 2020^25^	149	61±12.6	46	3	104	0.4	P	24±2.2	137±17
Raphael 2019^14^	194	67±12	67	3	30 28	0.8 0.5	P	24±2	127±13
Raphael 2020^26^	62	72±8	97	3	24	0.5	P	24±2	128±12
Witham 2020^19^	300	74±7.1	71	3	104	0.3	P	20.4±2.6	143±18

P, placebo; SC, standard care.

While systolic BP data were available for all trials, the data regarding antihypertensive and diuretic medication varied. Seven trials specified the number of patients who had increases in antihypertensive and diuretic medications during the follow-up period. Five trials provided further details regarding the increase and decrease in antihypertensive and diuretic medications, which were broken down by drug class (*e.g.*, renin-angiotensin-aldosterone system, *β*-blockers). Two studies collected detailed medication data, but these data were not made available because of the ongoing nature of these studies.^20,21^ Fully characterized drug data, *i.e.*, descriptive information of individual drug types within each class and the extent of dose change, were not available.

### Risk of Bias

Using the Cochrane Risk of Bias 2.0 assessment tool,^15^ nine of the 14 studies included in this review were evaluated as having a low risk of bias,^14,16,18,19,22–26^ four were identified to have some concerns (medium risk),^17,20,27,28^ and one study was identified as high risk^29^ (Supplemental Figure 1). Risk of bias mostly stemmed from the randomization process, bias due to deviations from intended interventions, and bias in selection of the reported result.

### Change in Mean Systolic BP

**Figure 2:**
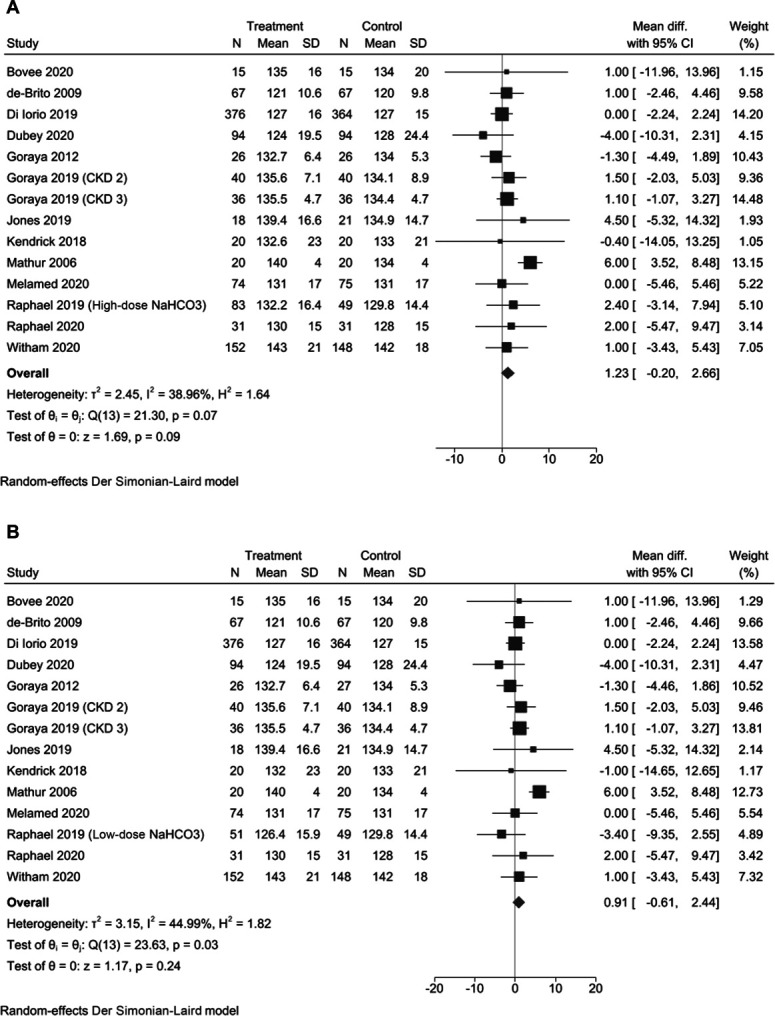
**Meta-analysis of systolic BP in (A) group A and (B) group B.** CI, confidence interval.

Fourteen trials (2110 participants) reported systolic BP data for end of intervention. We generated two meta‐analyses per outcome (to avoid double counting control group data): group A included 13 studies and the Raphael *et al.* (2019)^14^ high dose intervention arm data (*n*=2059), while group B included 13 studies and the Raphael *et al.* (2019)^14^ low dose intervention arm data (*n*=2027). Heterogeneity as quantified by I^2^ ranged from 39% to 45% for groups A and B, respectively. Heterogeneity was not significant for group A (*P* = 0.07) and significant for group B (*P* = 0.03), as defined using the χ^2^ statistic. Random-effects meta-analyses on the two groups did not show a statistical difference in systolic BP compared with a placebo or standard care (group A: standardized mean difference [SMD] 1.23 [95% CI, −0.20 to 2.60], *P* = 0.09 [Figure 2A], and group B: SMD 0.91 [95% CI, −0.61 to 2.44], *P* = 0.24 [Figure 2B]). Grading of Recommendations, Assessment, Development and Evaluations (GRADE) evaluation^30^ suggests that there is moderate certainty in these results (Supplemental Table 1).

### Sensitivity Analysis

We performed a sensitivity analysis excluding the study with high risk of bias,^29^ and the results remained consistent (group A: SMD 0.50 [95% CI, −0.58 to 1.58], *P* = 0.37 [I^2^ 0%, χ^2^ *P* = 0.94] [Supplemental Figure 2A], group B: SMD 0.29 [95% CI, −0.80 to 1.37], *P* = 0.60 [I^2^ 0%, χ^2^ *P* = 0.89] [Supplemental Figure 2B]). The results also remained consistent when excluding studies where a placebo was not used (Supplemental Figures 3 and 4), where participants were not acidotic at baseline (Supplemental Figure 5), where systolic BP was not a primary or secondary outcome (Supplemental Figure 6), or if dosing of sodium bicarbonate was adjusted by bodyweight (10 studies, group A: SMD 1.13 [95% CI, −0.73 to 2.99], *P* = 0.24, group B: SMD 0.68 [95% CI, −1.23 to 2.59], *P* = 0.49) or absolute dosing (four studies, SMD 1.24 [95% CI, −1.33 to 3.81], *P* = 0.34) (Supplemental Figure 7).

### Subgroup Analysis

**Figure 3:**
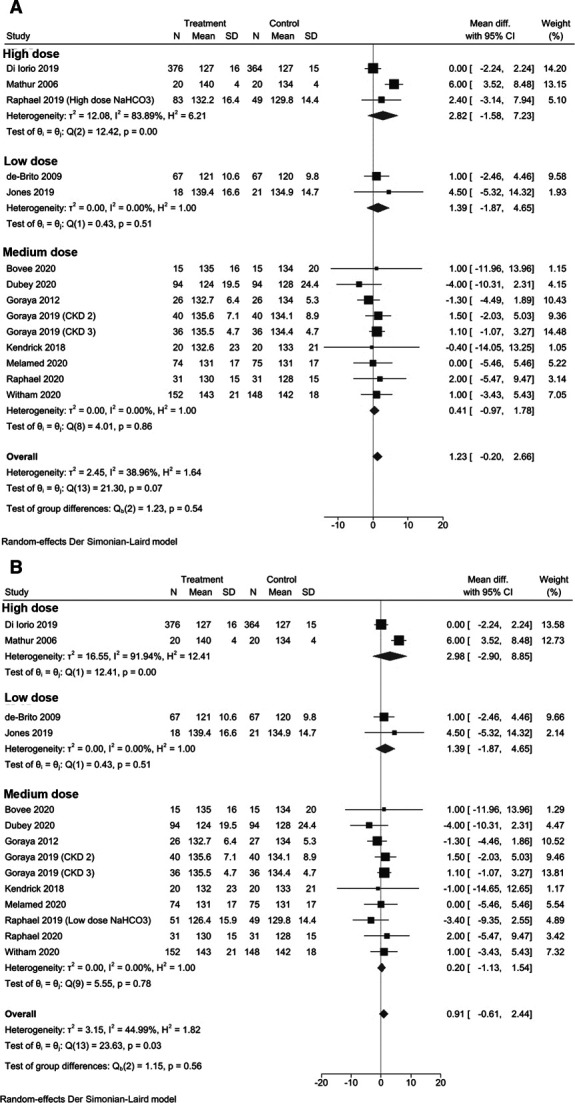
Subgroup analyses of systolic BP by (A) dose of sodium bicarbonate in group A and (B) dose of sodium bicarbonate in group B.

Subgroup analyses were conducted by dose of sodium bicarbonate (Figure 3), study duration (Supplemental Figure 8), and stage of CKD (Supplemental Figure 9), and the results remained consistent, with no significant differences in BP identified between sodium bicarbonate and control groups in any of the meta-analyses. Furthermore, meta-regression analysis did not identify any confounders to evaluate.

### Small Study Effect Bias

Small study effect bias was evaluated for groups A and B using funnel plot analysis (Supplemental Figure 10) and tested using an Egger test. Only the study with high risk of bias^29^ was outside the 95% CI. Studies were spread within the funnel plot area, and Egger values were *P* = 0.70 and *P* = 0.48 for groups A and B, respectively, suggesting that small study effect bias did not influence our results.

### Change in Mean Serum Bicarbonate

Change in mean serum bicarbonate was evaluated as a surrogate of sodium bicarbonate therapy adherence. High levels of heterogeneity (group A: I^2^ 96%, χ^2^ *P* < 0.05; group B I^2^ 96%, χ^2^ *P* < 0.05) excluded evaluation of the data using a forest plot, and sensitivity analyses did not significantly change this finding.

### Change in BP Management Medications

Medication data were available for all studies with the exception of two studies.^20,23^ However, in written and oral communication with the authors of these studies, it was reported that there was no significant difference in the use of antihypertensives and diuretics between the control and sodium bicarbonate intervention groups. Medication data collection varied: For some studies, it was an outcome of interest, while for others, it was collated as adverse event monitoring.

The analysis was separated for antihypertensive medication and diuretics. Antihypertensive medication data extracted fell into three main categories, studies where data were collated for (*1*) an increase in overall medication only, (*2*) both increases and decreases in overall medication, and (*3*) increases and decreases in medications grouped by drug class, *i.e.*, renin-angiotensin-aldosterone system, *β*-blockers, vasodilators, and “other” (including moxonidine, hydralazine, methyldopa). Each drug change event represents an empirical change due to a lack of available information regarding specific dose changes and the inability to compare doses of one antihypertensive medication with another. Owing to the complexity of the drug data collected, it was not evaluated by stage of CKD or dose of sodium bicarbonate. Furthermore, small study bias was not evaluated because of the presence of <8 studies in the analysis.

### Change in Antihypertensive Medications

#### Increase in All Antihypertensives

Heterogeneity values of I^2^ 73% and χ^2^ *P* < 0.0003 prevented meta-analysis in this sample. Sensitivity analysis did not change this result.

#### Decrease in Antihypertensives

**Figure 4:**
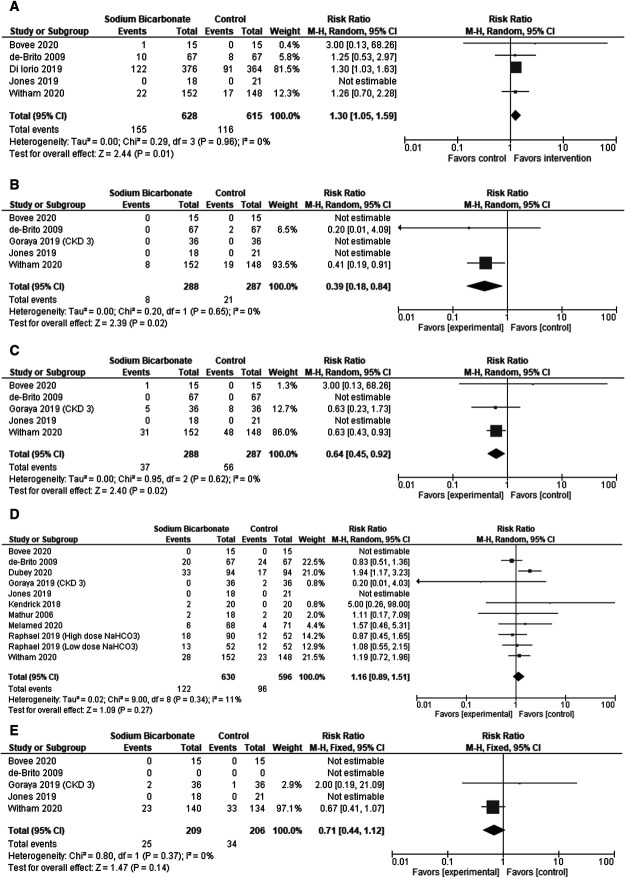
**Meta-analyses of antihypertensive medication change.** (A) Decrease in antihypertensive medications. (B) Increase in *β*-blockers. (C) Increase in vasodilators. (D) Increase in diuretic medications. (E) Decrease in diuretic medications.

Only five studies (575 participants) reported data for a decrease in the dose of antihypertensives. The meta-analysis suggests that there was a greater decrease in antihypertensive medication use in the sodium bicarbonate intervention group when compared with the control group: RR 1.30 (1.05–1.59), *P* < 0.01 (Figure 4A).

#### Changes by Drug Group

Five studies (575 participants) reported data for changes by drug group. When analyzed, these data suggest that the control group was more likely to have an increase in *β*-blocker and vasodilator use (RR 0.39 [0.18–0.84], *P* = 0.02 and RR 0.64 [0.45–0.92], *P* = 0.02, respectively) (Figure 4, B and C). There were no significant differences in the use of the renin-angiotensin-aldosterone system and “other” drugs.

#### Change in Diuretics

There were no significant differences in the use of diuretics between the control and sodium bicarbonate intervention groups (Figure 4, D and E).

## Discussion

In this systematic review and meta-analysis evaluating the effect of oral sodium bicarbonate on systolic BP in patients with CKD, which includes the evaluation of increases and decreases in antihypertensive medication change, we report that sodium bicarbonate does not increase systolic BP or the requirement for antihypertensive medication or diuretics. Sensitivity analyses and subgroup analyses support this finding.

These findings are consistent with previous meta-analyses. Hultin and colleagues^10^ assessed the influence of sodium bicarbonate on systolic BP in 1932 individuals across 12 studies and reported no significant differences in systolic BP between intervention and control groups; however, in a subanalysis of five RCTs (1383 individuals), the researchers noted a significant worsening of systolic BP in the intervention arm. Similarly, Navaneethan *et al.*^12^ reported on seven studies, including 711 individuals, and demonstrated no significant increase in systolic BP (SMD 20.1 [21.9–1.7], *P* = 0.93), with a small increase in systolic BP reported in a meta-analysis of three studies (362 individuals, RR 1.38 [1.07–1.79], *P* = 0.01). By contrast, Cheng *et al.*^11^ reported that sodium bicarbonate reduced systolic BP by 2.97 mm Hg (−5.04 to −0.90) in a meta-analysis of six studies, including 1312 individuals, although there were some concerns regarding the heterogeneity in those studies (χ^2^= *P* = 0.04, I^2^=56%).

Regarding changes in dosage of antihypertensive medication and diuretics as a surrogate to systolic BP change, no other meta-analysis has attempted to evaluate this in detail. Navaneethan *et al.*^12^ found a positive association between sodium bicarbonate therapy and both antihypertensive medication and diuretics. However, there was inherent bias in the one-way analysis performed because it did not evaluate any decreases in therapy. Understanding bidirectional change in antihypertensive medication is important because it may enable future stratification of individuals who are more likely to benefit from sodium bicarbonate therapy to manage metabolic acidosis and as an adjunct to systolic BP management strategies. Our data suggest that sodium bicarbonate therapy is not associated with an overall increase in antihypertensive medication or diuretic therapy. In fact, our results suggest that the control group was more likely to have an increase in *β*-blockers and vasodilators (Figure 4, B and D). However, it should be noted that GRADE evaluation highlights uncertainty in the antihypertensive medication and diuretic findings, due to the difficulty of comparing dose changes for antihypertensive medication and diuretics within and between the included trials.

Clinical guidance regarding the use of sodium bicarbonate in CKD is limited and variable. KDIGO guidance^31^ states that with “serum bicarbonate concentrations <22 mmol/L, treatment with oral bicarbonate supplementation be given to maintain serum bicarbonate within the normal range unless contraindicated.” These contraindications relate to the concerns regarding the sodium load associated with sodium bicarbonate therapy. These concerns stem from three key sources: (*1*) the paucity of RCTs, specifically evaluating the effect of sodium bicarbonate on systolic BP as a primary end point, (*2*) from an understanding of the role of sodium in renin-angiotensin-aldosterone system activation, and (*3*) overwhelming evidence demonstrating that sodium chloride increases systolic BP.^8^

Evidence to challenge the concern that sodium bicarbonate loading increases systolic BP in CKD exists in small-scale, nonrandomized studies.^9,32–34^ These studies also suggest that in “normal” participants and participants with CKD, sodium ingested in the form of bicarbonate is fully excreted in urine, whereas sodium in the form of chloride is retained, enhancing its effect on systolic BP.

As a surrogate of sodium bicarbonate therapy, we explored serum bicarbonate after therapy. However, we note the high heterogeneity in this analysis. This intriguing finding can be a sign of poor adherence to the intervention, perhaps because of medication tolerance, need for up-titration of sodium bicarbonate to counterbalance the ongoing intake of high acid-containing Western diets,^35^ or interindividual variability in response to sodium bicarbonate. Indeed, high interindividual variability has been shown across a number of independent studies^36,37^ and requires more evaluation.

This systematic review and meta-analysis is strengthened by several factors. We included a larger number of trials compared with previous meta-analyses, spanning more than 2000 participants. We used stringent inclusion criteria limited to RCTs and performed risk-of-bias assessment using the Cochrane Risk of Bias 2.0 tool.^15^ We also conducted several subgroup analyses and meta-regression to ascertain the role of potential confounders. However, we also acknowledge some limitations. First, systolic BP and/or antihypertensive medication change was not a primary or secondary outcome for a number of studies, and this can undermine confidence in the effect measured. Moreover, treatment allocation is not randomized, thus antihypertensive medication analyses are prone to confounding by indication. Second, there was a paucity of RCTs that account for bidirectional changes in medication data. Third, the eligibility criteria of the included studies excluded individuals with uncontrolled high BP, and we are unable to comment on the effects on participants with severe or moderately uncontrolled hypertension. Fourth, the granularity of data when conducting a systematic review and meta-analysis prevents us from truly accounting for interindividual variability. As discussed above, more research is needed in this area. Finally, the medication data retrieved is also heterogeneous, presenting a possible residual confounding factor that we cannot account for.

In the first systematic review and meta-analysis of sodium bicarbonate, BP, and metabolic acidosis to account for changes in medication, we find that sodium bicarbonate supplementation does not change systolic BP. Consequently, clinical hesitation for the use of sodium bicarbonate in participants with CKD seems unwarranted. We also show no effect when stratifying our analysis by dose administered or duration of intervention. More effort is needed within the CKD–sodium bicarbonate field to record thorough data regarding changes in medication from intervention.

## Supplementary Material

## Supplemental Material

Published online ahead of print. Publication date available athttp://links.lww.com/CJN/B644.

Supplemental Methods.

Supplemental Table 1. GRADE summary of findings.

Supplemental Figure 1. Risk of bias assessment summary for included studies.

Supplemental Figure 2. Sensitivity analysis: Panel A: Excluding studies with high risk of bias for systolic BP (SBP) in group A and Panel B: Excluding studies with high risk of bias in group B.

Supplemental Figure 3. Sensitivity analysis of SBP excluding studies where a placebo was not used in group A (Panel A) and group B (Panel B).

Supplemental Figure 4. Sensitivity analysis of SBP: Panel A: Excluding studies where a placebo was not used (excluding studies with high risk of bias) in group A. Panel B: Excluding studies where a placebo was not used (excluding studies with high risk of bias) in group B.

Supplemental Figure 5. Panel A: Sensitivity analysis excluding studies where participants were not acidotic at study entry. Panel B: Sensitivity analysis excluding studies where participants were not acidotic at study entry (excluding high risk of bias study).

Supplemental Figure 6. Sensitivity analysis excluding studies where SBP was not a primary or secondary outcome.

Supplemental Figure 7. Sensitivity analysis by weight-based dosing (group A: Panel A, group B: Panel B) or absolute dosing (Panel C) of sodium bicarbonate.

Supplemental Figure 8. Subgroup analysis by length of study for SBP in group A (Panel A) and group B (Panel B).

Supplemental Figure 9. Subgroup analysis of SBP by (A) stage of CKD in group A and (B) stage of CKD in group B.

Supplemental Figure 10. Funnel plot to evaluate small study bias in SBP in group A (Panel A) and group B (Panel B).
