# Supplementary material for: Effect of Sodium Bicarbonate on Systolic Blood Pressure in CKD: A Systematic Review and Meta-Analysis
Source: Clin J Am Soc Nephrol. 2023 Mar 9;18(4):435–45. doi: 10.2215/CJN.0000000000000119 (PMC10103210; doi:10.2215/CJN.0000000000000119)

Supplemental Material: Effect of Sodium Bicarbonate on Systolic Blood Pressure in CKD: A Systematic Review and Meta-Analysis

Beverley Beynon-Cobb[1,2,#,*], Panayiotis Louca[2,*], Ewout J. Hoorn[4], Cristina Menni[2,#,†], Sandosh Padmanabhan[3,†]

1. Department of Nutrition & Dietetics, University Hospitals Coventry & Warwickshire NHS Trust, Coventry, CV2 2DX, United Kingdom
2. Department of Twin Research and Genetic Epidemiology, King's College London, London, England, SE1 7EH, United Kingdom.
3. Institute of Cardiovascular & Medical Sciences, University of Glasgow, Glasgow G12 8QQ, United Kingdom.
4. Department of Internal Medicine, Divisions of Nephrology and Transplantation, Erasmus Medical Center, University Medical Center Rotterdam, Room Ns403, PO Box 2040, 3000, CA, Rotterdam, The Netherlands

# Corresponding author
* equal contribution
† equal contribution

Supplemental Methods
Supplemental Table 1: GRADE Summary of Findings
Supplemental Figure 1: Risk of Bias Assessment summary for included studies
Supplemental Figure 2: Sensitivity analysis: Panel A: Excluding high risk of bias studies for SBP in Group A: Panel B: Excluding high risk of bias studies in Group B.
Supplemental Figure 3: Sensitivity analysis of SBP excluding studies where a placebo was not used in Group A (Panel A) Group B (Panel B).
Supplemental Figure 4: Sensitivity analysis of SBP: Panel A: excluding studies where a placebo was not used (excluding high risk of bias studies) in Group A. Panel B: excluding studies where a placebo was not used (excluding high risk of bias studies) in Group B.
Supplemental Figure 5: Panel A: Sensitivity analysis excluding studies where subjects were not acidotic at study entry: Panel B: Sensitivity analysis excluding studies where subjects were not acidotic at study entry (excluding high risk of bias study).
Supplemental Figure 6: Sensitivity analysis excluding studies where SBP was not a primary or secondary outcome.
Supplemental Figure 7: Sensitivity analysis by weight-based dosing (group A: panel A, group B: panel B) or absolute dosing (panel C) of sodium bicarbonate.
Supplemental Figure 8: Sub-group analysis by length of study for Systolic Blood Pressure in group A (Panel A) and group B (Panel B).
Supplemental Figure 9: Sub-group analysis of Systolic blood pressure by A: Stage of CKD in Group A. B: Stage of CKD in Group B.
Supplemental Figure 10: Funnel plot to evaluate small study bias in SBP in group A (Panel A) and group B (Panel B).

Supplemental Methods

Search Strings in literature databases.

Medline search strings

S72 s34 NOT s71

S71 S35 OR S36 OR S37 OR S38 OR S39 OR S40 OR S41 OR S42 OR S43 OR S44 OR S45 OR S46 OR S47 OR S48 OR S49 OR S50 OR S51 OR S52 OR S53 OR S54 OR S55 OR S56 OR S57 OR S58 OR S59 OR S60 OR S61 OR S62 OR S63 OR S64 OR S65 OR S66 OR S67 OR S68 OR S69 OR S70

S70 TI Renal bone disease OR AB Renal bone disease

S69 TI nephrocalcinosis OR AB nephrocalcinosis

S68 (MH "Nephrocalcinosis")

S67 TI Cystin* OR AB Cystin*

S66 TI Cystinosis OR AB Cystinosis

S65 (MH "Cystinuria")

S64 TI hyperoxaluria OR AB hyperoxaluria

S63 TI calcul* OR AB calcul*

S62 (MH "Kidney Calculi+")

S61 TI Contrast OR AB Contrast

S60 (MH "Contrast Media+")

S59 TI haemofiltration OR AB haemofiltration

S58 TI Hemofiltration OR AB Hemofiltration

S57 (MH "Hemofiltration+")

S56 TI Peritoneal dialysis OR AB Peritoneal dialysis

S55 TI haemodialysis OR AB haemodialysis

S54 TI Hemodialysis OR AB Hemodialysis

S53 TI Dialysis OR AB Dialysis

S52 TI Renal dialysis OR AB Renal dialysis

S51 TI Renal replacement therapy OR AB Renal replacement therapy

S50 (MH "Renal Replacement Therapy+")

S49 TI Dialysate OR AB Dialysate

S48 (MH "Dialysis Solutions")

S47 TI Dialysis solutions OR AB Dialysis solutions

S46 TI Critical care OR AB Critical care

Search modes - Boolean/Phrase

S45 (MH "Critical Care+")

S44 TI Intensive care unit OR AB Intensive care unit

S43 TI Intensive therapy unit OR AB Intensive therapy unit

S42 (MH "Intensive Care Units+")

S41 TI neonat* OR AB neonat*

S40 (MH "Infant, Newborn+")

S39 TI Pregnan* OR AB Pregnan*

S38 TI Pregnancy OR AB Pregnancy

S37 TI acute renal OR AB acute renal

S36 TI Acute kidney OR AB Acute kidney

S35 (MH "Kidney Failure, Acute+")

S34 S20 AND S32

S33 S20 AND S32

S32 S21 OR S22 OR S23 OR S24 OR S25 OR S26 OR S27 OR S28 OR S29 OR S30 OR S31

S31 TI Alkali therapy OR AB Alkali therapy

S30 TI Acidosis correction OR AB Acidosis correction

S29 TI Acidosis treatment OR AB Acidosis treatment

S28 TI Baking soda OR AB Baking soda

S27 TI Na citrate OR AB Na citrate

S26 TI NaHCO3 OR AB NaHCO3

S25 TI Bicarbonate OR AB bicarbonate

S24 TI Sodium citrate OR AB Sodium citrate

S23 (MH "Sodium Citrate")

S22 TI Sodium bicarbonate OR AB Sodium bicarbonate

S21 (MH "Sodium Bicarbonate+")

S20 S1 OR S2 OR S3 OR S4 OR S5 OR S6 OR S7 OR S8 OR S9 OR S10 OR S11 OR S12 OR S13 OR S14 OR S15 OR S16 OR S17 OR S18 OR S19

S19 TI Pre-dialysis OR AB Pre-dialysis Search modes

S18 TI Kidney disease progression OR AB Kidney disease progression

S17 TI Renal disease progression OR AB Renal disease progression

S16 TI Creatinine clearance OR AB Creatinine clearance

S15 TI GFR OR AB GFR

S14 TI Glomerular filtration rate OR AB Glomerular filtration rate

S13 (MH "Glomerular Filtration Rate")

S12 TI Renal function OR AB Renal function

S11 (MH "Kidney Function Tests+")

S10 TI Renal Disease OR AB Renal Disease

S9 TI Chronic Renal disease OR AB Chronic Renal disease

S8 TI Kidney failure OR AB Kidney failure

S7 TI Renal failure OR AB Renal failure

S6 TI Chronic renal failure OR AB Chronic renal failure

S5 TI Renal insufficiency OR AB Renal insufficiency

S4 TI Chronic renal insufficiency OR AB Chronic renal insufficiency

S3 TI Kidney disease OR AB Kidney disease

S2 TI Chronic kidney disease OR AB Chronic kidney disease

S1 (MH "Kidney Failure, Chronic+")

Cinahl search strings

S34 NOT S71

S71 S35 OR S36 OR S37 OR S38 OR S39 OR S40 OR S41 OR S42 OR S43 OR S44 OR S45 OR S46 OR S47 OR S48 OR S49 OR S50 OR S51 OR S52 OR S53 OR S54 OR S55 OR S56 OR S57 OR S58 OR S59 OR S60 OR S61 OR S62 OR S63 OR S64 OR S65 OR S66 OR S67 OR S68 OR S69 OR S70

Search modes -

S70 TI Renal bone disease OR AB Renal bone disease

S69 TI nephrocalcinosis OR AB nephrocalcinosis

S68 (MH "Nephrocalcinosis")

S67 TI Cystin* OR AB Cystin*

S66 TI Cystinosis OR AB Cystinosis

S65 (MH "Cystinuria")

S64 TI hyperoxaluria OR AB hyperoxaluria

S63 TI calcul* OR AB calcul*

S62 (MH "Kidney Calculi+")

S61 TI Contrast OR AB Contrast

S60 (MH "Contrast Media+")

S59 TI haemofiltration OR AB haemofiltration

S58 TI Hemofiltration OR AB Hemofiltration

S57 (MH "Hemofiltration+")

S56 TI Peritoneal dialysis OR AB Peritoneal dialysis

S55 TI haemodialysis OR AB haemodialysis

S54 TI Hemodialysis OR AB Hemodialysis

S53 TI Dialysis OR AB Dialysis

S52 TI Renal dialysis OR AB Renal dialysis

S51 TI Renal replacement therapy OR AB Renal replacement therapy

S50 (MH "Renal Replacement Therapy+")

S49 TI Dialysate OR AB Dialysate

S48 (MH "Dialysis Solutions")

S47 TI Dialysis solutions OR AB Dialysis solutions

S46 TI Critical care OR AB Critical care

S45 (MH "Critical Care+")

S44 TI Intensive care unit OR AB Intensive care unit

S43 TI Intensive therapy unit OR AB Intensive therapy unit

S42 (MH "Intensive Care Units+")

S41 TI neonat* OR AB neonat*

S40 (MH "Infant, Newborn+")

S39 TI Pregnan* OR AB Pregnan*

S38 TI Pregnancy OR AB Pregnancy

S37 TI acute renal OR AB acute renal

S36 TI Acute kidney OR AB Acute kidney

S35 (MH "Kidney Failure, Acute+")

S34 S20 AND S32

S33 S20 AND S32

S32 S21 OR S22 OR S23 OR S24 OR S25 OR S26 OR S27 OR S28 OR S29 OR S30 OR S31 Search

S31 TI Alkali therapy OR AB Alkali therapy

S30 TI Acidosis correction OR AB Acidosis correction

S29 TI Acidosis treatment OR AB Acidosis treatment

S28 TI Baking soda OR AB Baking soda

S27 TI Na citrate OR AB Na citrate

S26 TI NaHCO3 OR AB NaHCO3

S25 TI Bicarbonate OR AB bicarbonate

S24 TI Sodium citrate OR AB Sodium citrate

S23 (MH "Sodium Citrate")

S22 TI Sodium bicarbonate OR AB Sodium bicarbonate

S21 (MH "Sodium Bicarbonate+")

S20 S1 OR S2 OR S3 OR S4 OR S5 OR S6 OR S7 OR S8 OR S9 OR S10 OR S11 OR S12 OR S13 OR S14 OR S15 OR S16 OR S17 OR S18 OR S19

S19 TI Pre-dialysis OR AB Pre-dialysis

S18 TI Kidney disease progression OR AB Kidney disease progression

S17 TI Renal disease progression OR AB Renal disease progression

S16 TI Creatinine clearance OR AB Creatinine clearance

S15 TI GFR OR AB GFR

S14 TI Glomerular filtration rate OR AB Glomerular filtration rate

S13 (MH "Glomerular Filtration Rate")

S12 TI Renal function OR AB Renal function

S11 (MH "Kidney Function Tests+")

S10 TI Renal Disease OR AB Renal Disease

S9 TI Chronic Renal disease OR AB Chronic Renal disease

S8 TI Kidney failure OR AB Kidney failure

S7 TI Renal failure OR AB Renal failure

S6 TI Chronic renal failure OR AB Chronic renal failure

S5 TI Renal insufficiency OR AB Renal insufficiency

S4 TI Chronic renal insufficiency OR AB Chronic renal insufficiency

S3 TI Kidney disease OR AB Kidney disease

S2 TI Chronic kidney disease OR AB Chronic kidney disease

S1 (MH "Kidney Failure, Chronic+")

AMED search strings

1. **exp Kidney disease/ or exp Kidney failure chronic/**
2. **(Chronic adj kidney adj disease).ti.**
3. **(Chronic adj kidney adj disease).ab.**
4. **(renal adj insufficiency).ti.**
5. **(renal adj insufficiency).ab.**
6. **(renal adj failure).ab.**
7. **(renal adj failure).ti.**
8. **(Kidney adj failure).ti.**
9. **(Kidney adj failure).ab.**
10. **(Renal adj disease).ab.**
11. **(Renal adj disease).ti.**
12. **(Kidney adj disease).ab.**
13. **(Kidney adj function).ab.**
14. **(Kidney adj function).ti.**
15. **(Renal adj function).ti.**
16. **(Renal adj function).ab.**
17. **(Glomerular adj filtration adj rate).ti.**
18. **(Glomerular adj filtration adj rate).ab.**
19. **GFR.ab.**
20. **GFR.ti.**
21. **(Creatinine adj clearance).ti.**
22. **(Creatinine adj clearance).ab.**
23. **Pre-dialysis.ab.**
24. **Pre-dialysis.ti.**

25. 1 or 2 or 3 or 4 or 5 or 6 or 7 or 8 or 9 or 10 or 11 or 12 or 13 or 14 or 15 or 16 or 17 or 18 or 19 or 20 or 21 or 22 or 23 or 24

26. (Sodium adj bicarbonate).ti.

27. (Sodium adj bicarbonate).ab.

28. Bicarbonate.ab.

29. Bicarbonate.ti.

30. NaHCO3.ti.

31. NaHCO3.ab.

32. (Sodium adj Citrate).ab.

33. (Sodium adj Citrate).ti.

34. (Baking adj soda).ti.

35. (Baking adj soda).ab.

36. (Acidosis adj2 treatment).ab.

37. (Acidosis adj2 treatment).ti.

38. (Acidosis adj2 correction).ti.

39. (Acidosis adj2 correction).ab.

40. (Alkali adj therapy).ab.

41. (Alkali adj therapy).ti.

42. 26 or 27 or 28 or 29 or 30 or 31 or 32 or 33 or 34 or 35 or 36 or 37 or 38 or 39 or 40 or 41

EMBASE search strings

1. **exp chronic kidney failure/**
2. **(Chronic adj kidney adj disease).ab.**
3. **(Chronic adj kidney adj disease).ti.**
4. **(renal adj insufficiency).ab.**
5. **renal insufficiency.ti.**
6. **(renal adj failure).ti.**
7. **(renal adj failure).ab.**
8. **(Kidney adj failure).ab.**
9. **(Kidney adj failure).ti.**
10. **(Renal adj disease).ti.**
11. **(Renal adj disease).ab.**
12. **(Kidney adj disease).ab.**
13. **(Kidney adj disease).ti.**
14. **exp kidney function/**
15. **(Kidney adj function).ti.**
16. **(Kidney adj function).ab.**
17. **(Renal adj function).ab.**
18. **(Renal adj function).ti.**
19. **exp glomerulus filtration rate/**
20. **(Glomerular adj filtration adj rate).ti.**
21. **(Glomerular adj filtration adj rate).ab.**
22. **GFR.ab. or GFR.ti.**
23. **exp creatinine clearance/**
24. **(Creatinine adj clearance).ab.**
25. **(Creatinine adj clearance).ti.**

26. Pre-dialysis.ti. or Pre-dialysis.ab.

27. 1 or 2 or 3 or 4 or 5 or 6 or 7 or 8 or 9 or 10 or 11 or 12 or 13 or 14 or 15 or 16 or 17 or 18 or 19 or 20 or 21 or 22 or 23 or 24 or 25 or 26

28. exp bicarbonate/

29. (Sodium adj bicarbonate).ti.

30. (Sodium adj bicarbonate).ab.

31. Bicarbonate.ab. or Bicarbonate.ti.

32. NaHCO3.ab. or NaHCO3.ti.

33. exp citrate sodium/

34. (Sodium adj Citrate).ab.

35. (Sodium adj Citrate).ti.

36. (Baking adj soda).ti.

37. (Baking adj soda).ab.

38. (Acidosis adj treatment).ab.

39. (Acidosis adj treatment).ti.

40. (Acidosis adj2 treatment).ti.

41. (Acidosis adj2 treatment).ab.

42. (Acidosis adj2 correction).ab.

43. (Acidosis adj2 correction).ti.

44. (Alkali adj therapy).ti.

45. (Alkali adj therapy).ab.

46. 28 or 29 or 30 or 31 or 32 or 33 or 34 or 35 or 36 or 37 or 38 or 39 or 40 or 41 or 42 or 43 or 44 or 45

47. 27 and 46

48. exp acute kidney failure/

49. (Acute adj kidney).ab.

50. (Acute adj kidney).ti.

51. (Acute adj renal).ti.

52. (Acute adj renal).ab.

53. exp pregnancy/

54. pregnan*.ab. or pregnan*.ti.

55. newborn/

56. Neonat*.ab. or Neonat*.ti.

57. exp intensive care unit/

58. (Intensive adj therapy adj unit).ab.

59. (Intensive adj therapy adj unit).ti.

60. (Intensive adj care adj unit).ti.

61. (Intensive adj care adj unit).ab.

62. (Critical adj care).ab.

63. (Critical adj care).ti.

64. exp dialysis fluid/

65. (Dialysis adj solutions).ti.

66. (Dialysis adj solutions).ab.

67. (dialysis adj fluid).ab.

68. (dialysis adj fluid).ti.

69. Dialysate.ti. or Dialysate.ab.

70. exp renal replacement therapy/

71. (Renal adj replacement).ti.

72. (Renal adj replacement).ab.

73. Dialysis.ab. or Dialysis.ti.

74. Haemodialysis.ab. or Haemodialysis.ti.

75. hemodialysis.ab. or hemodialysis.ti.

76. (Peritoneal adj dialysis).ab.

77. (Peritoneal adj dialysis).ti.

78. exp hemofiltration/

79. exp continuous hemofiltration/

80. Hemofiltration.ti. or Hemofiltration.ab.

81. haemofiltration.ti. or haemofiltration.ab.

82. exp contrast sensitivity/ or exp contrast induced nephropathy/ or exp contrast/

83. Contrast.ti. or Contrast.ab.

84. exp nephrolithiasis/

85. stones.ti. or stones.ab.

86. exp hyperoxaluria/

87. hyperoxaluria.ti. or hyperoxaluria.ab.

88. exp cystinuria/

89. exp cystinosis/

90. Cystin*.ti. or Cystin*.ab.

91. calcul*.ti. or calcul*.ab.

92. exp renal osteodystrophy/

93. (Renal adj bone adj disease).ti.

94. (Renal adj bone adj disease).ab.

95. 48 or 49 or 50 or 51 or 52 or 53 or 54 or 55 or 56 or 57 or 58 or 59 or 60 or 61 or 62 or 63 or 64 or 65 or 66 or 67 or 68 or 69 or 70 or 71 or 72 or 73 or 74 or 75 or 76 or 77 or 78 or 79 or 80 or 81 or 82 or 83 or 84 or 85 or 86 or 87 or 88 or 89 or 90 or 91 or 92 or 93 or 94

96. (((chronic kidney failure or (Chronic adj kidney adj disease) or (Chronic adj kidney adj disease) or (renal adj insufficiency) or renal insufficiency or (renal adj failure) or (renal adj failure) or (Kidney adj failure) or (Kidney adj failure) or (Renal adj disease) or (Renal adj disease) or (Kidney adj disease) or (Kidney adj disease) or kidney function or (Kidney adj function) or

(Kidney adj function) or (Renal adj function) or (Renal adj function) or glomerulus filtration rate or (Glomerular adj filtration adj rate) or (Glomerular adj filtration adj rate) or (GFR or GFR) or creatinine clearance or (Creatinine adj clearance) or (Creatinine adj clearance) or (Pre-dialysis or Pre-dialysis)) and (bicarbonate or (Sodium adj bicarbonate) or (Sodium adj bicarbonate) or (Bicarbonate or Bicarbonate) or (NaHCO3 or NaHCO3) or citrate sodium or (Sodium adj Citrate) or (Sodium adj Citrate) or (Baking adj soda) or (Baking adj soda) or (Acidosis adj treatment) or (Acidosis adj treatment) or (Acidosis adj2 treatment) or (Acidosis adj2 treatment) or (Acidosis adj2 correction) or (Acidosis adj2 correction) or (Alkali adj therapy) or (Alkali adj therapy))) not (acute kidney failure or (Acute adj kidney) or (Acute adj kidney) or (Acute adj renal) or (Acute adj renal) or pregnancy or (pregnan* or pregnan*) or newborn or (Neonat* or Neonat*) or intensive care unit or (Intensive adj therapy adj unit) or (Intensive adj therapy adj unit) or (Intensive adj care adj unit) or (Intensive adj care adj unit) or (Critical adj care) or (Critical adj care) or dialysis fluid or (Dialysis adj solutions) or (Dialysis adj solutions) or (dialysis adj fluid) or (dialysis adj fluid) or (Dialysate or Dialysate) or renal replacement therapy or (Renal adj replacement) or (Renal adj replacement) or (Dialysis or Dialysis) or (Haemodialysis or Haemodialysis) or (hemodialysis or hemodialysis) or (Peritoneal adj dialysis) or (Peritoneal adj dialysis) or hemofiltration or continuous hemofiltration or (Hemofiltration or Hemofiltration) or (haemofiltration or haemofiltration) or (contrast sensitivity or contrast induced nephropathy or contrast) or (Contrast or Contrast) or nephrolithiasis or (stones or stones) or hyperoxaluria or (hyperoxaluria or hyperoxaluria) or cystinuria or cystinosis or (Cystin* or Cystin*) or (calcul* or calcul*) or renal osteodystrophy or (Renal adj bone adj disease) or (Renal adj bone adj disease))).ti.

Supplemental Table 1: GRADE Summary of Findings

Outcomes	Anticipated absolute effects[*] (95% CI)		Relative effect (95% CI)	No of participants (studies)	Certainty of the evidence (GRADE)
	Risk with placebo	Risk with Sodium bicarbonate			
End intervention SBP (Raphael High dose included) mmHg		MD 1.1 higher (0.59 higher to 1.61 higher)	-	2058 (14 RCTs)	⊕⊕⊕◯ Moderate[a]
End intervention SBP (Raphael Low dose included) mmHg		MD 1.01 higher (0.23 lower to 2.25 higher)	-	2026 (14 RCTs)	⊕⊕⊕◯ Moderate[a]
Antihypertensives decrease	189 per 1,000	245 per 1,000 (198 to 300)	RR 1.30 (1.05 to 1.59)	1243 (5 RCTs)	⊕◯◯◯ Very low[b,c]

**Increase B-Blocker**	**73 per 1,000**	**29 per 1,000 (13 to 61)**	**RR 0.39 (0.18 to 0.84)**	**575 (5 RCTs)**	**⊕◯◯◯ Very low[b,c]**
**Increase Vasodilator**	**195 per 1,000**	**125 per 1,000 (88 to 180)**	**RR 0.64 (0.45 to 0.92)**	**575 (5 RCTs)**	**⊕◯◯◯ Very low[b,c]**

Explanations

a. SBP was not a primary or secondary outcome of interest for many of the studies included which may affect the outcome of this review

b. Collation of drug data as a surrogate of SBP change was not a primary or secondary outcome of interest for studies included. Drug dose changes were not clarified to explain what constituted dose change in each study i.e., for the same drugs or for drugs within the same class.

c. The optimal information size is <2000

Supplemental Figure 1: Risk of Bias Assessment summary for included studies.

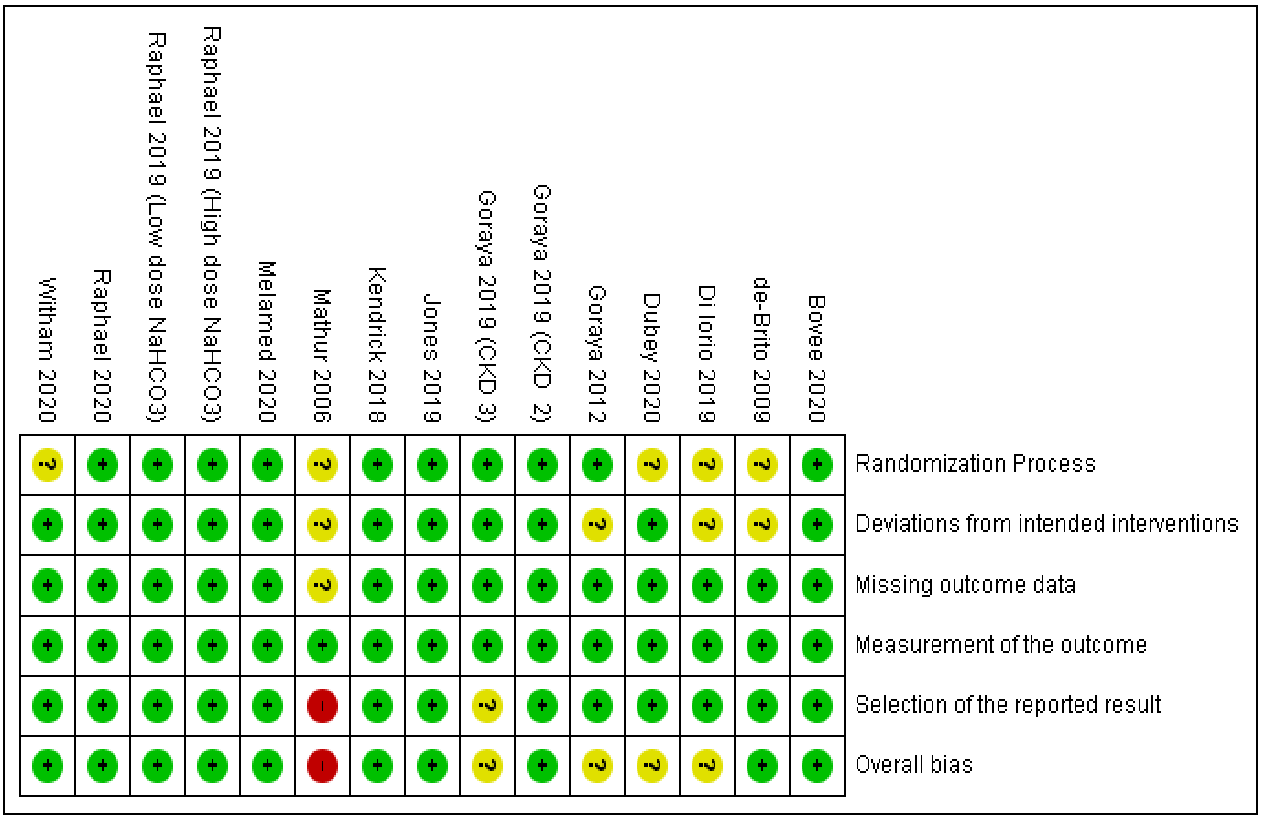

Supplemental Figure 2: Sensitivity analysis: Panel A: Excluding high risk of bias studies for SBP in Group A: Panel B: Excluding high risk of bias studies in Group B.

Panel A:

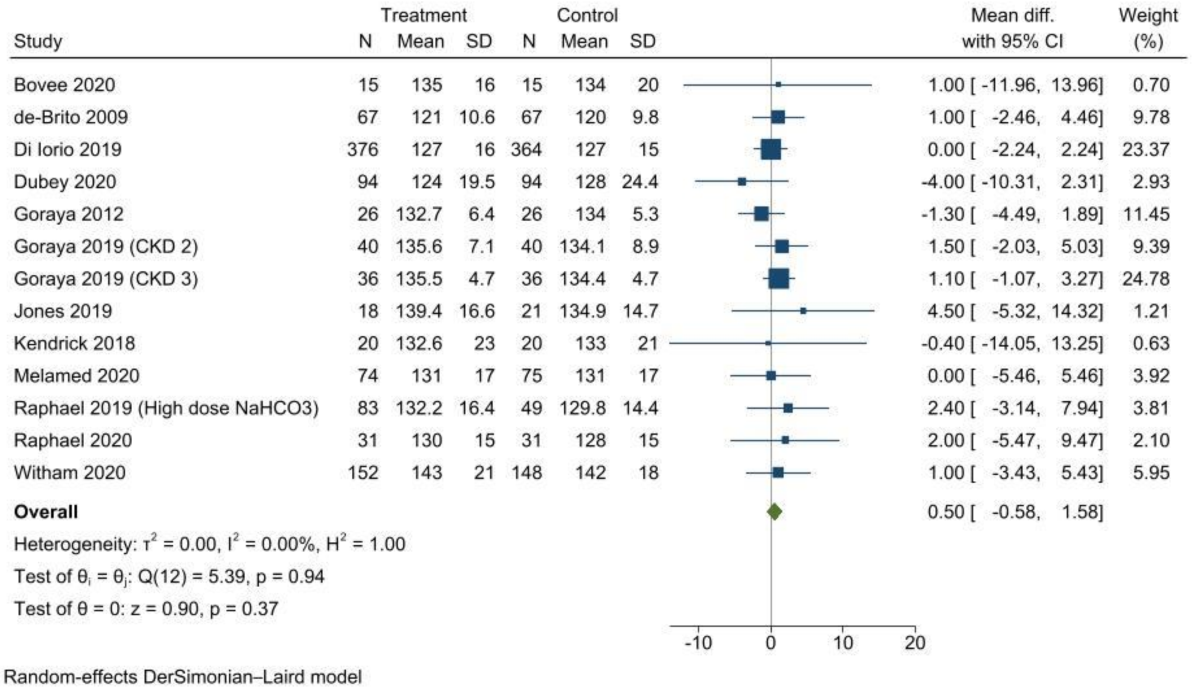

Panel B

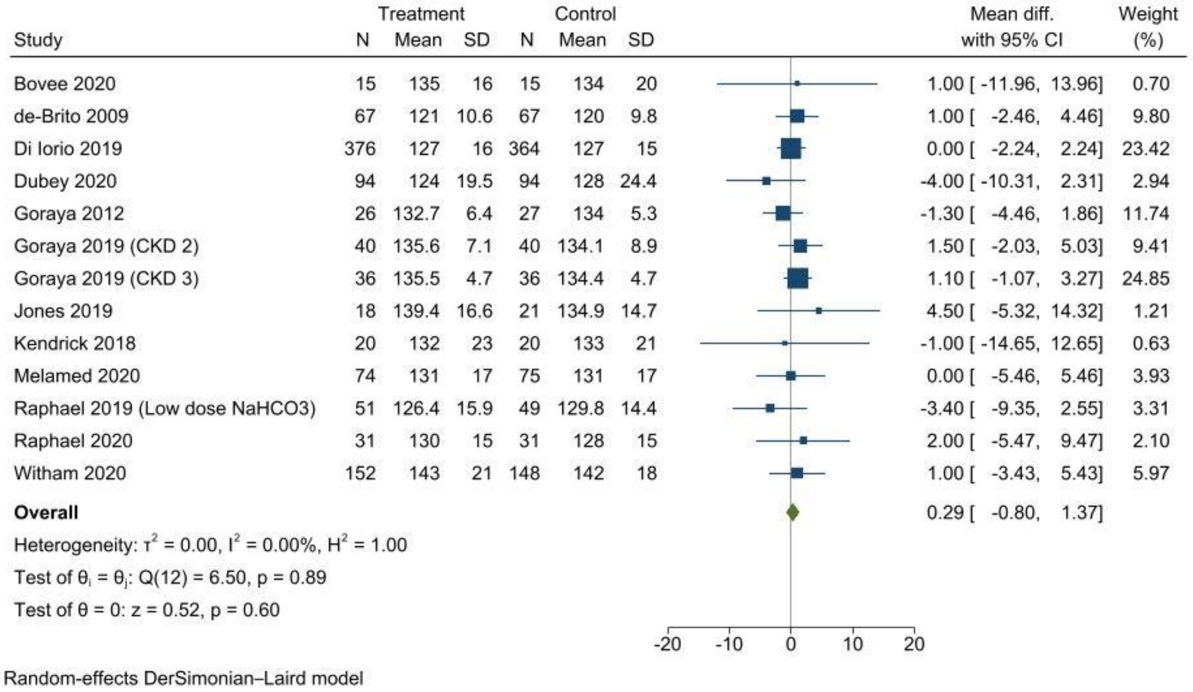

Supplemental Figure 3: Sensitivity analysis of SBP excluding studies where a placebo was not used in Group A (Panel A) Group B (Panel B).

Panel A

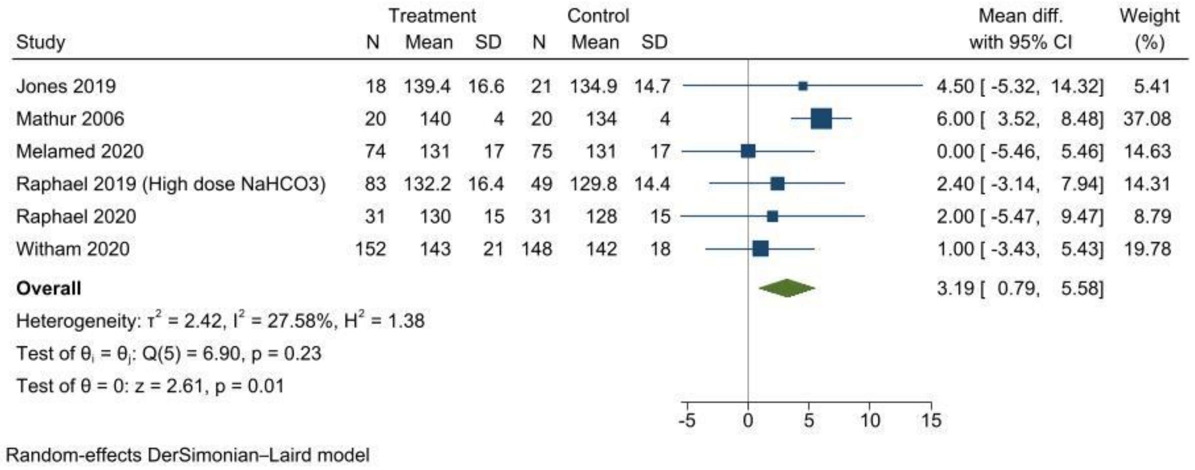

Panel B

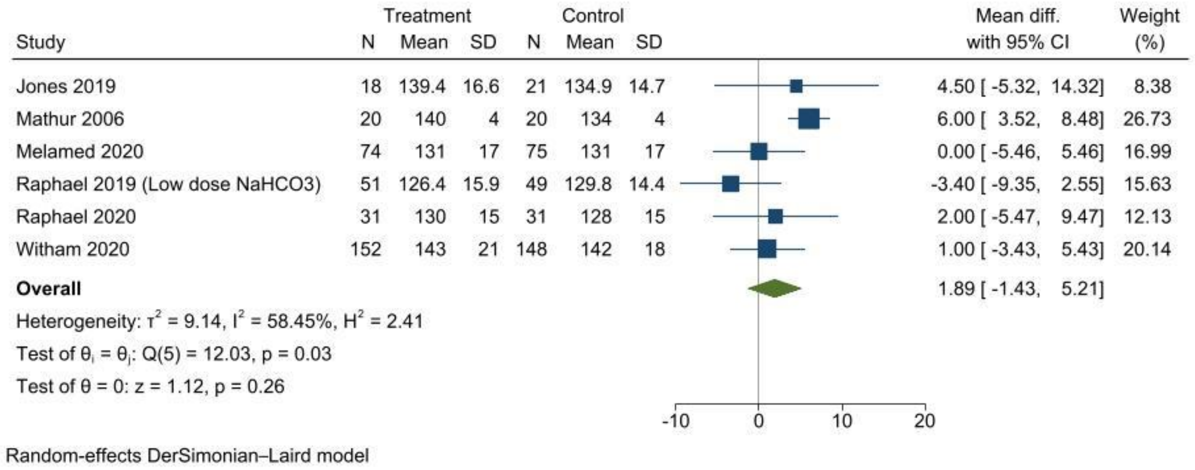

Supplemental Figure 4: Sensitivity analysis of SBP: Panel A: excluding studies where a placebo was not used (excluding high risk of bias studies) in Group A. Panel B: excluding studies where a placebo was not used (excluding high risk of bias studies) in Group B.

Panel A

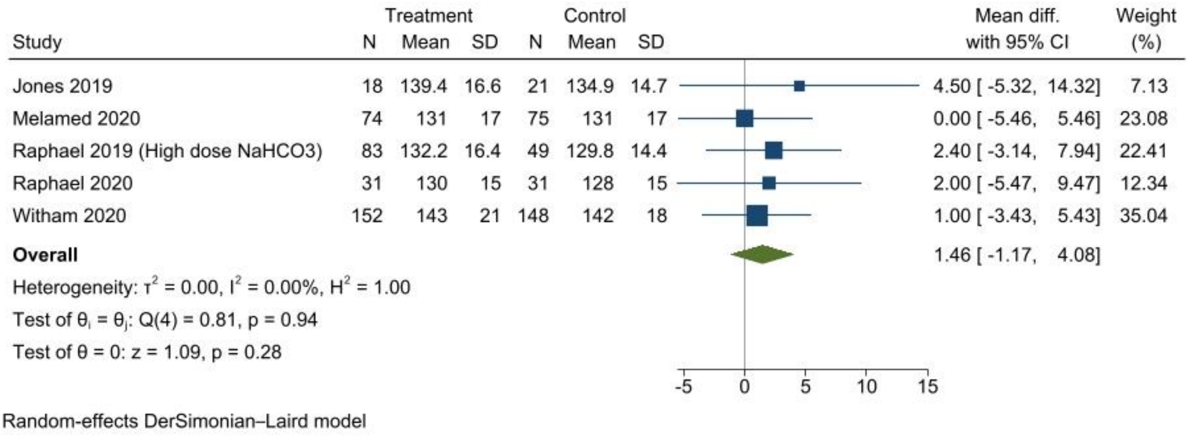

Panel B

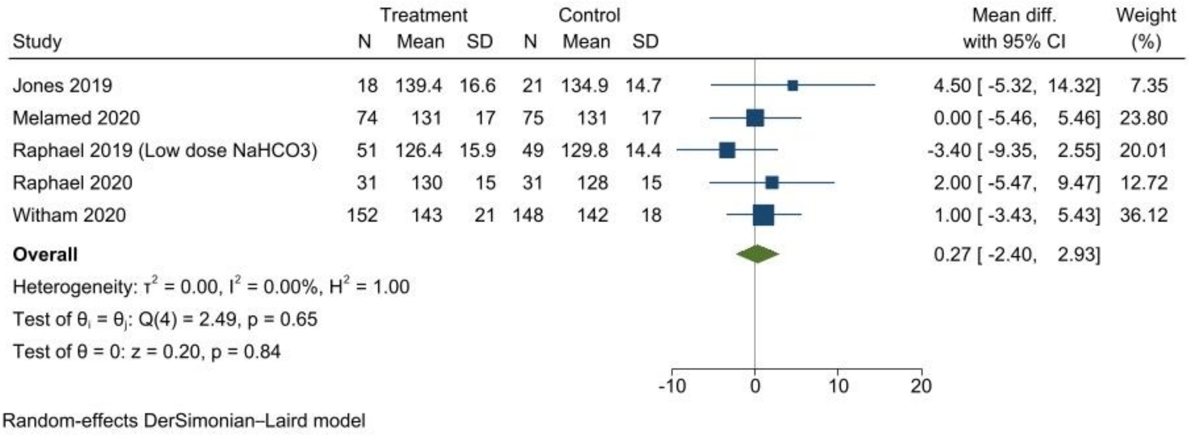

Supplemental Figure 5: Panel A: Sensitivity analysis excluding studies where subjects were not acidotic at study entry: Panel B: Sensitivity analysis excluding studies where subjects were not acidotic at study entry (excluding high risk of bias study).

Panel A

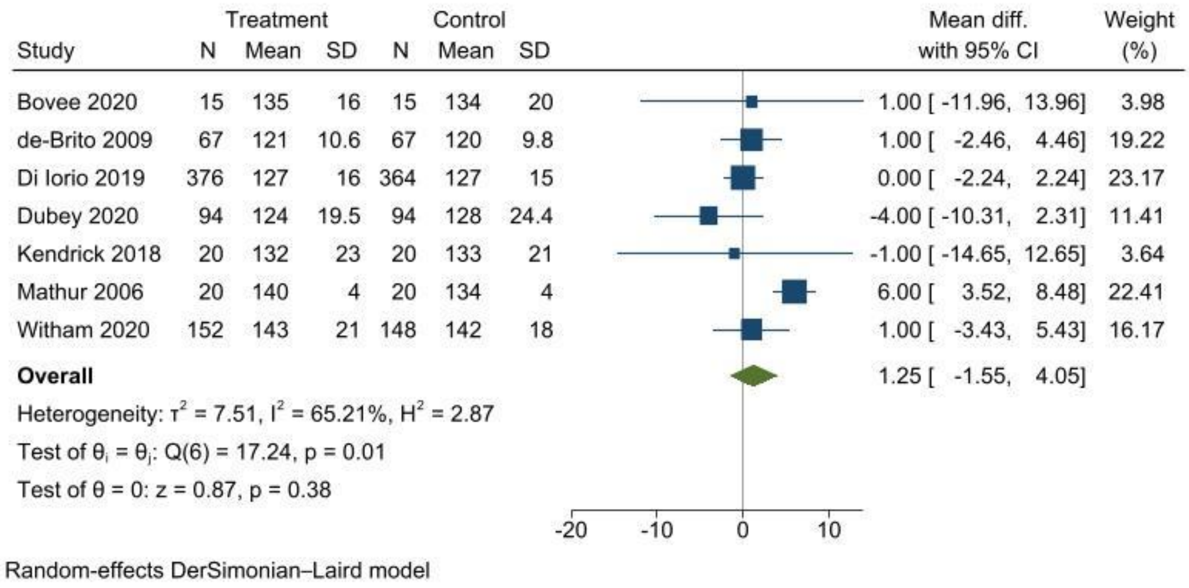

Panel B

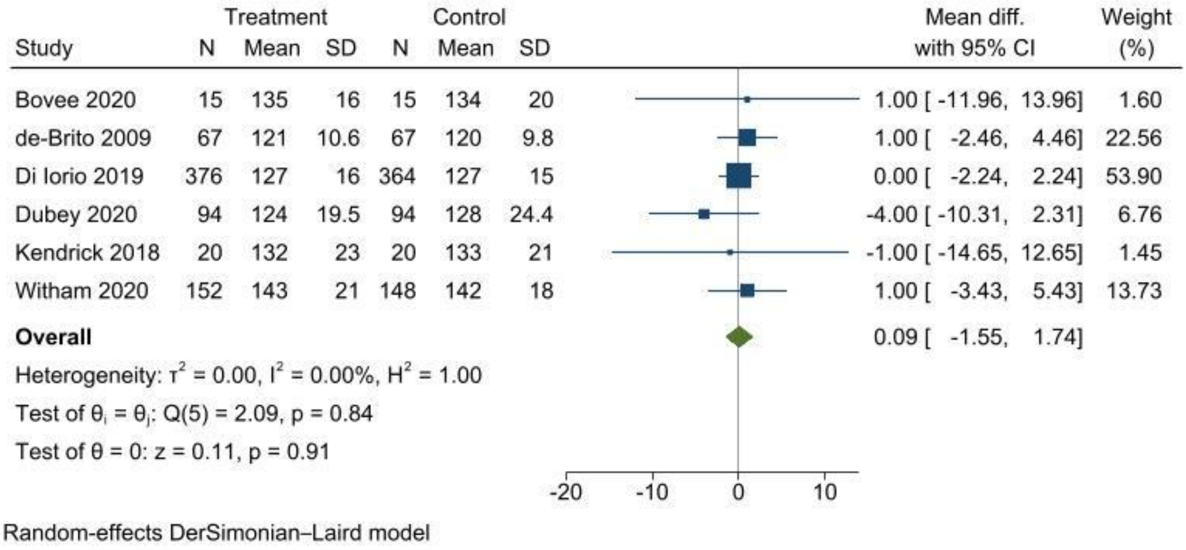

Supplemental Figure 6: Sensitivity analysis excluding studies where SBP was not a primary or secondary outcome.

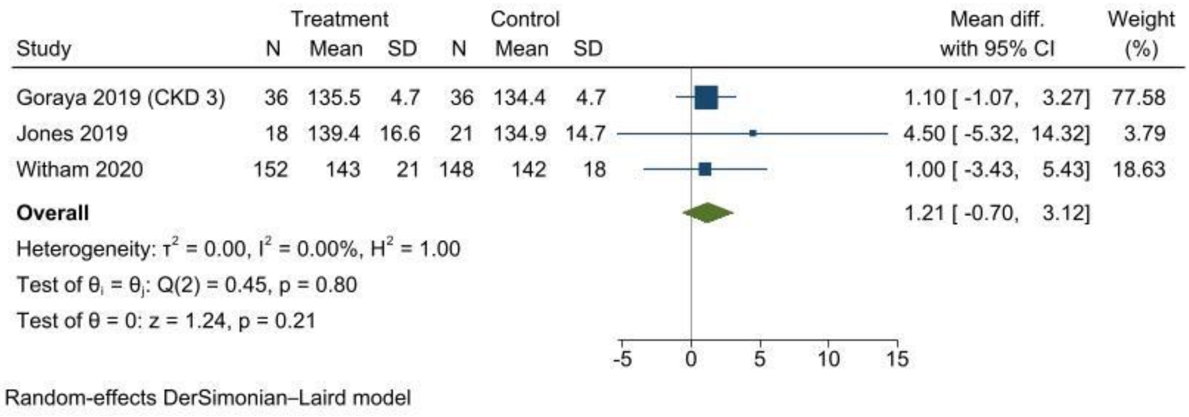

Supplemental Figure 7: Sensitivity analysis by weight-based dosing (group A: panel A, group B: panel B) or absolute dosing (panel C) of sodium bicarbonate.

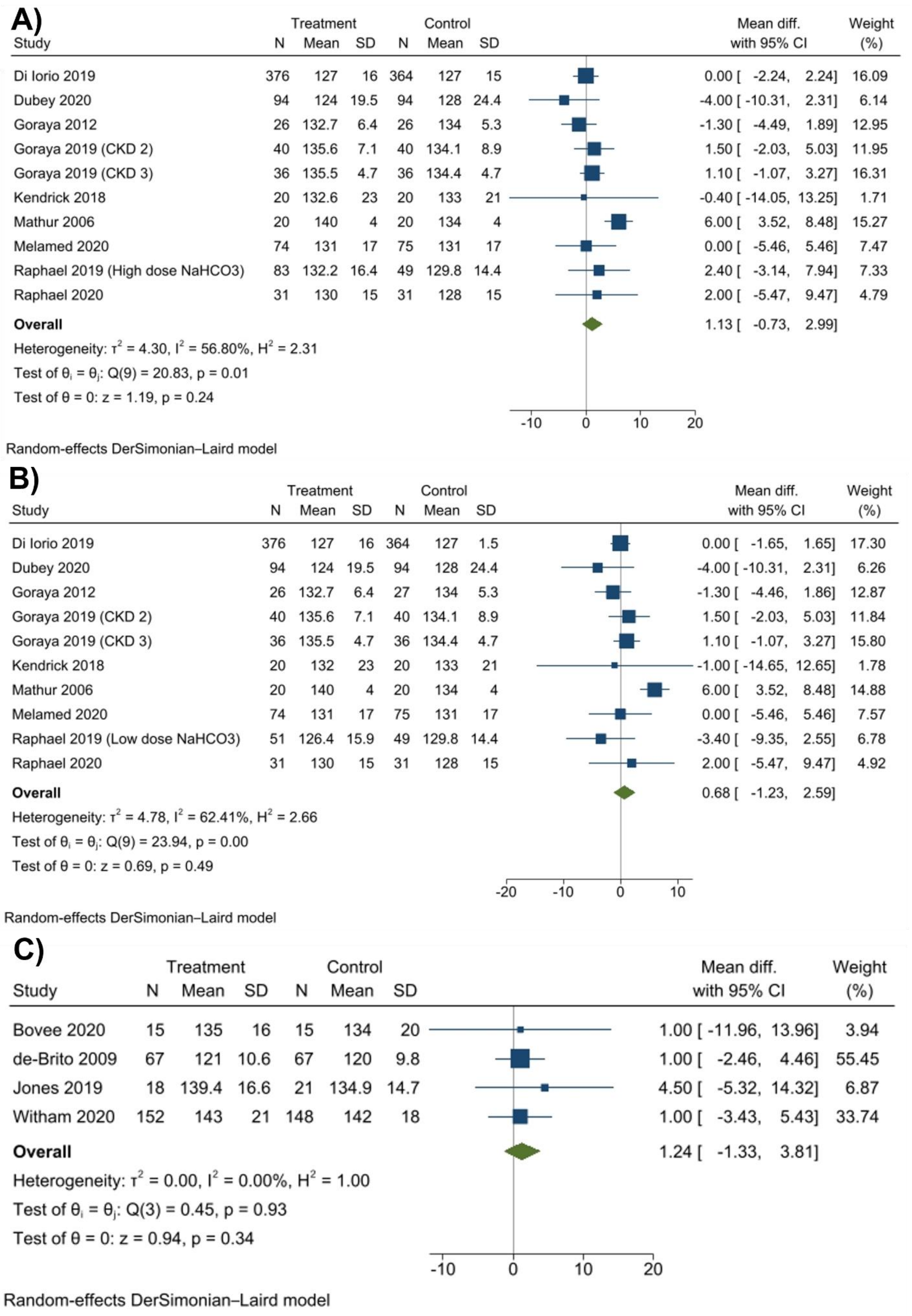

Supplemental Figure 8: Sub-group analysis by length of study for Systolic Blood Pressure in group A (Panel A) and group B (Panel B).

Panel A

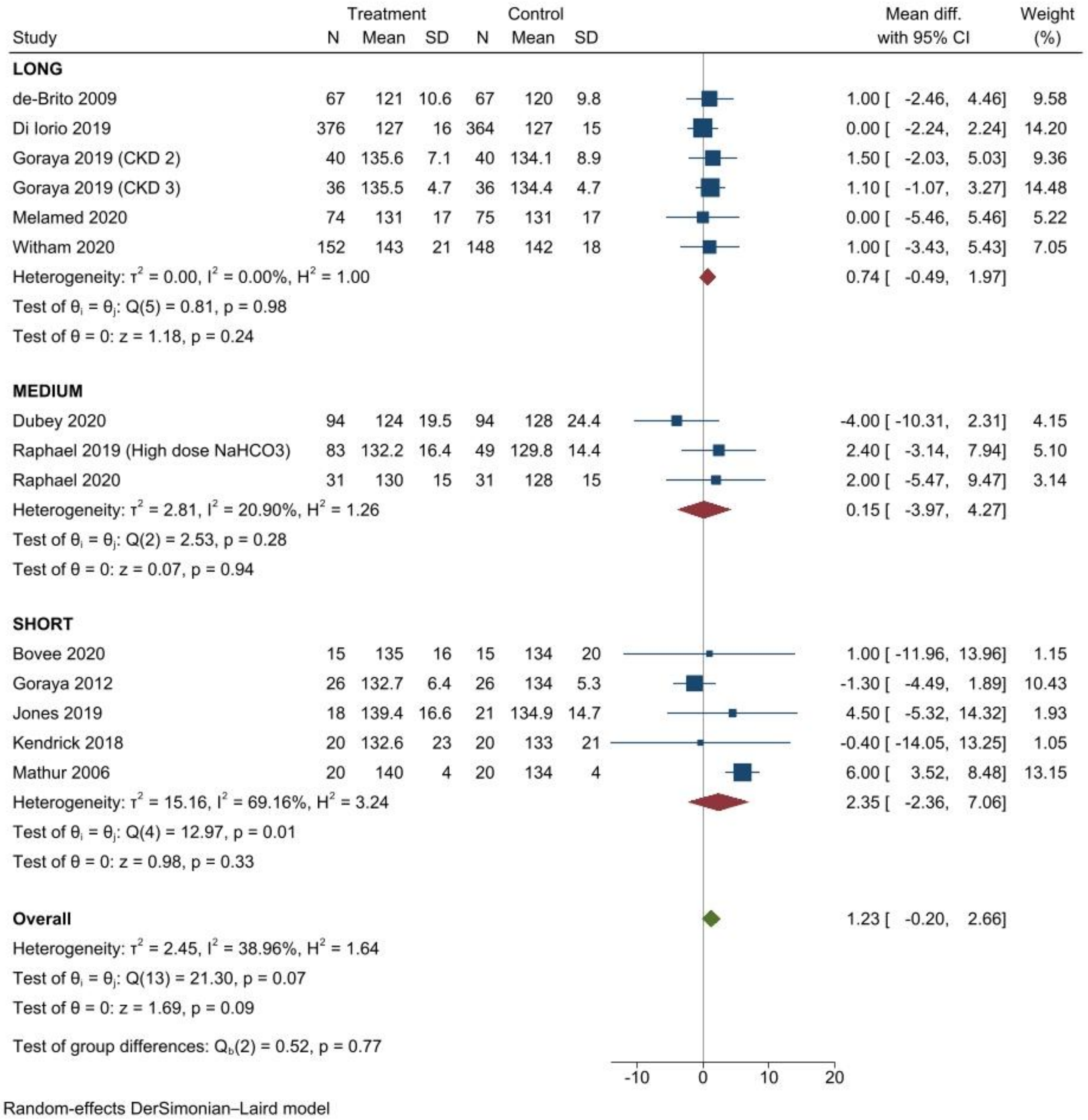

Panel B

Study	Treatment N	Treatment Mean	Treatment SD	Control N	Control Mean	Control SD	Mean diff. with 95% CI	Weight (%)
**LONG**								
de-Brito 2009	67	121	10.6	67	120	9.8	1.00 [-2.46, 4.46]	9.66
Di Iorio 2019	376	127	16	364	127	15	0.00 [-2.24, 2.24]	13.58
Goraya 2019 (CKD 2)	40	135.6	7.1	40	134.1	8.9	1.50 [-2.03, 5.03]	9.46
Goraya 2019 (CKD 3)	36	135.5	4.7	36	134.4	4.7	1.10 [-1.07, 3.27]	13.81
Melamed 2020	74	131	17	75	131	17	0.00 [-5.46, 5.46]	5.54
Witham 2020	152	143	21	148	142	18	1.00 [-3.43, 5.43]	7.32
Heterogeneity: $\tau^2 = 0.00$, $I^2 = 0.00\%$, $H^2 = 1.00$							0.74 [-0.49, 1.97]	
Test of $\theta_i = \theta_j$: Q(5) = 0.81, p = 0.98								
Test of $\theta = 0$: z = 1.18, p = 0.24								
**MEDIUM**								
Dubey 2020	94	124	19.5	94	128	24.4	-4.00 [-10.31, 2.31]	4.47
Raphael 2019 (Low dose NaHCO3)	51	126.4	15.9	49	129.8	14.4	-3.40 [-9.35, 2.55]	4.89
Raphael 2020	31	130	15	31	128	15	2.00 [-5.47, 9.47]	3.42
Heterogeneity: $\tau^2 = 0.00$, $I^2 = 0.00\%$, $H^2 = 1.00$							-2.25 [-6.00, 1.50]	
Test of $\theta_i = \theta_j$: Q(2) = 1.68, p = 0.43								
Test of $\theta = 0$: z = -1.18, p = 0.24								
**SHORT**								
Bovee 2020	15	135	16	15	134	20	1.00 [-11.96, 13.96]	1.29
Goraya 2012	26	132.7	6.4	27	134	5.3	-1.30 [-4.46, 1.86]	10.52
Jones 2019	18	139.4	16.6	21	134.9	14.7	4.50 [-5.32, 14.32]	2.14
Kendrick 2018	20	132	23	20	133	21	-1.00 [-14.65, 12.65]	1.17
Mathur 2006	20	140	4	20	134	4	6.00 [3.52, 8.48]	12.73
Heterogeneity: $\tau^2 = 15.44$, $I^2 = 69.78\%$, $H^2 = 3.31$							2.29 [-2.45, 7.02]	
Test of $\theta_i = \theta_j$: Q(4) = 13.23, p = 0.01								
Test of $\theta = 0$: z = 0.95, p = 0.34								
**Overall**							0.91 [-0.61, 2.44]	
Heterogeneity: $\tau^2 = 3.15$, $I^2 = 44.99\%$, $H^2 = 1.82$								
Test of $\theta_i = \theta_j$: Q(13) = 23.63, p = 0.03								
Test of $\theta = 0$: z = 1.17, p = 0.24								
Test of group differences: $Q_b(2) = 2.76$, p = 0.25								

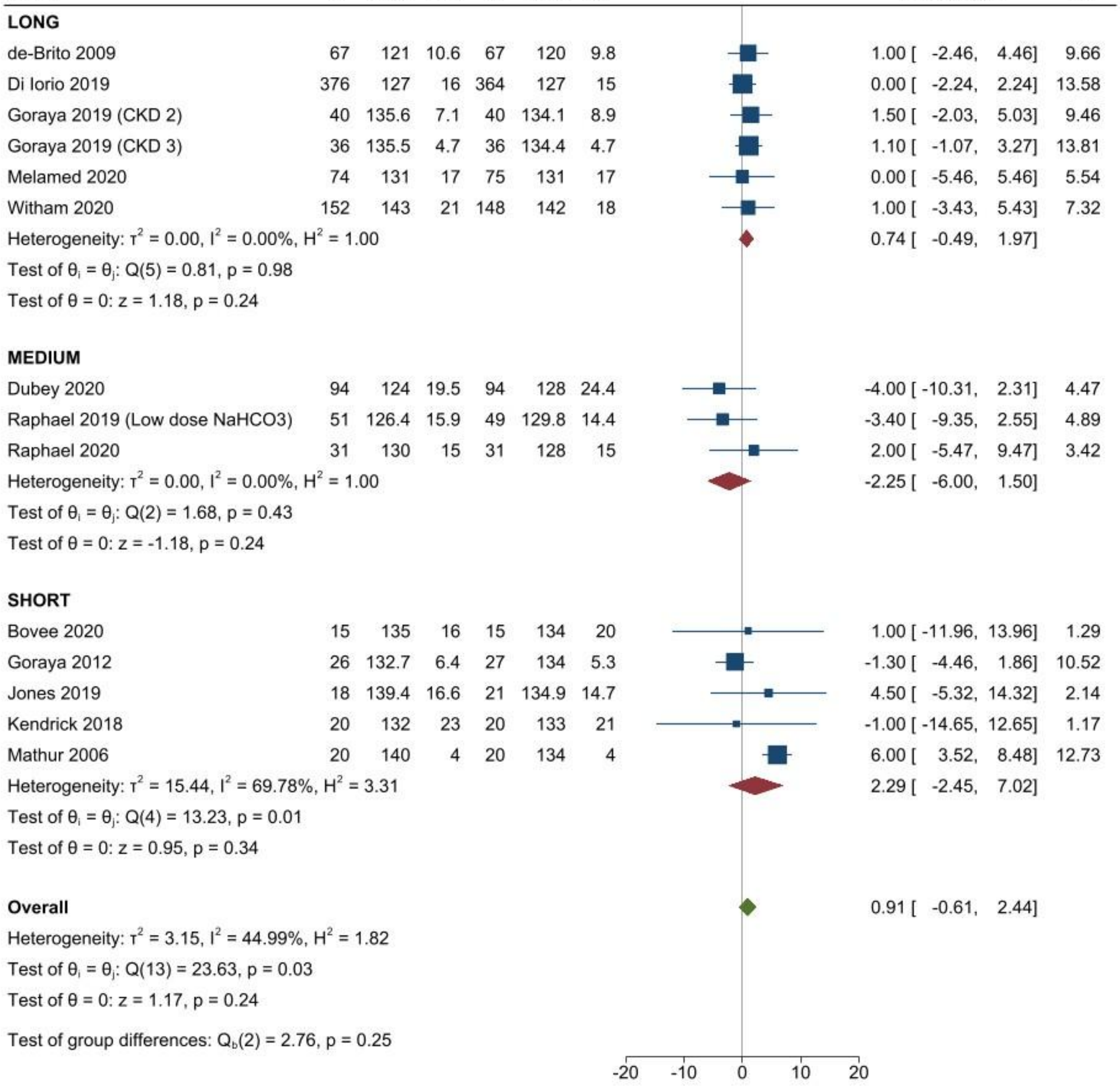

Random-effects DerSimonian–Laird model

Supplemental Figure 9: Sub-group analysis of Systolic blood pressure by A: Stage of CKD in Group A. B: Stage of CKD in Group B.

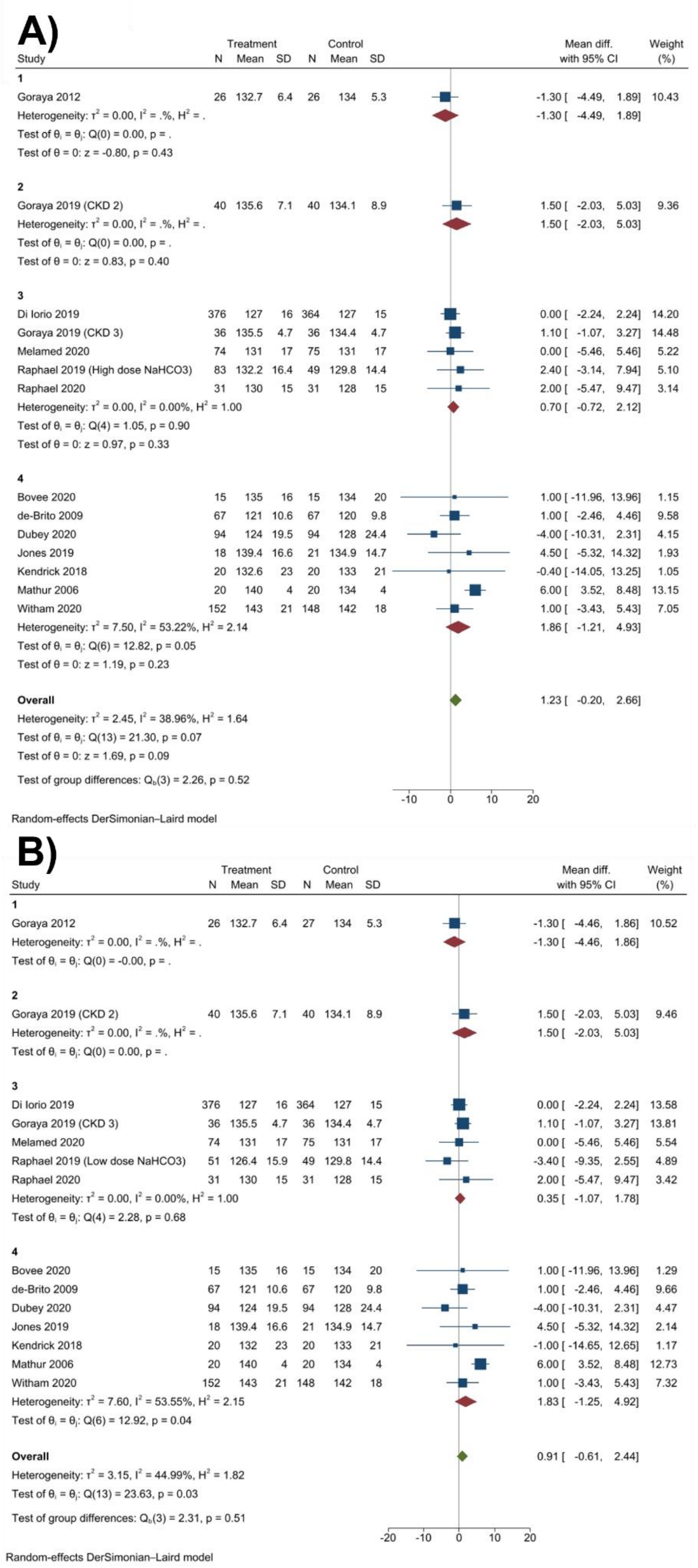

Supplemental Figure 10: Funnel plot to evaluate small study bias in SBP in group A (Panel A) and group B (Panel B).

Panel A

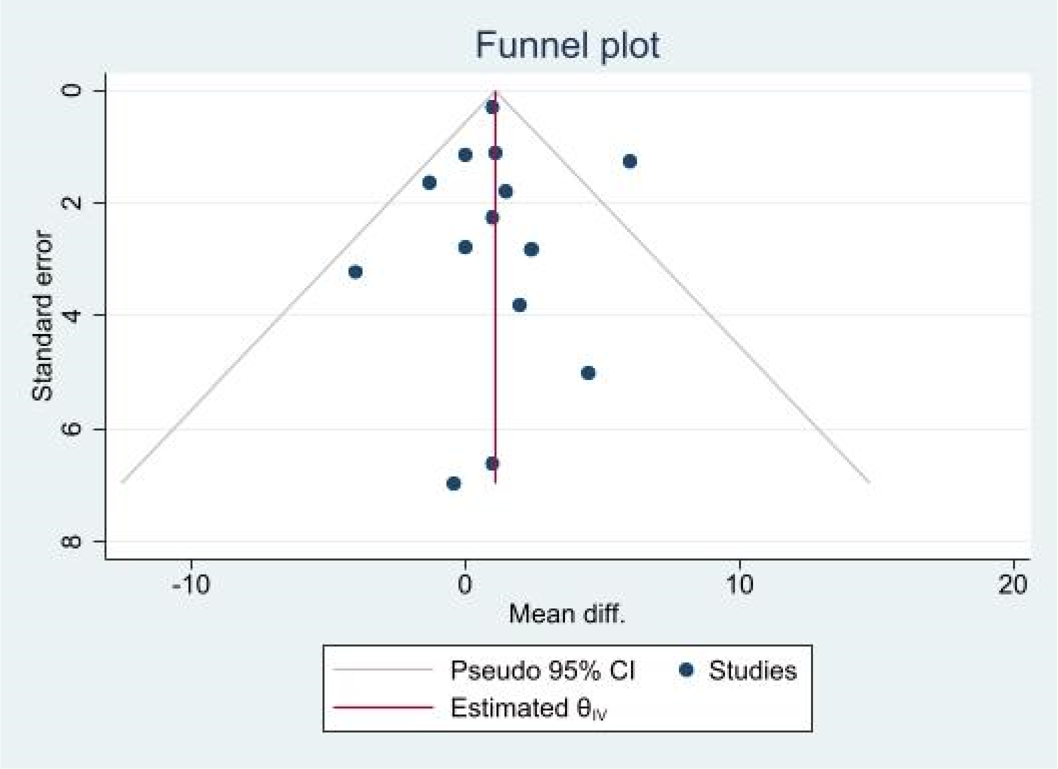

Panel B

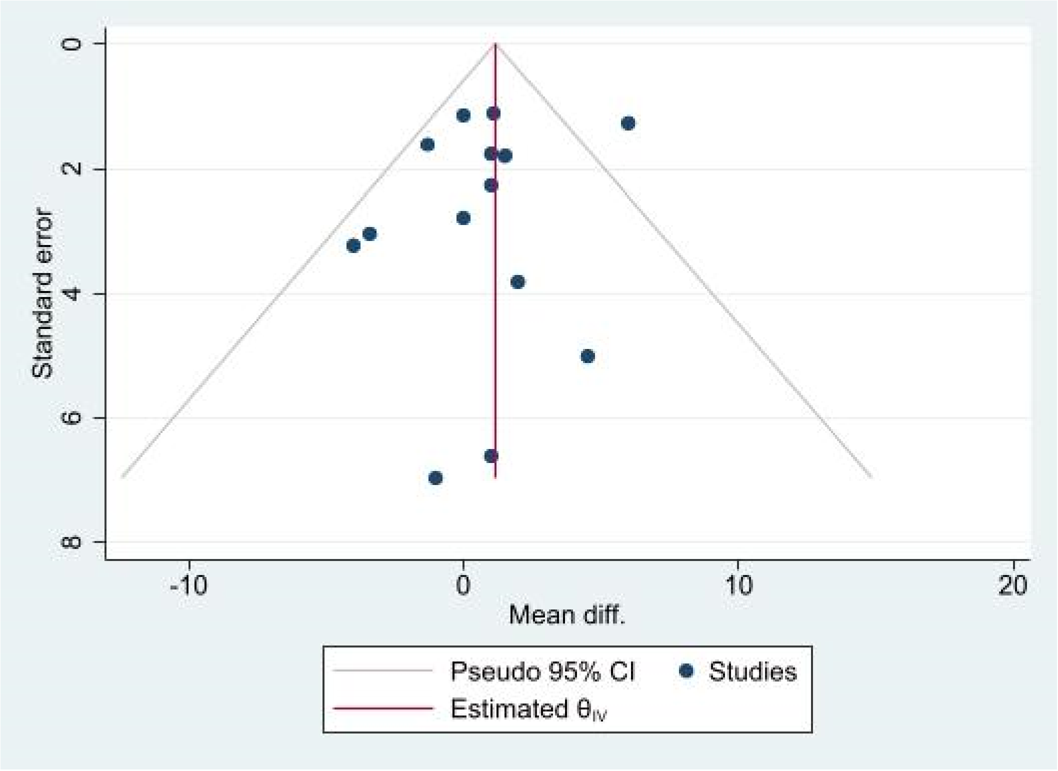

Supplement: Supplementary file 1 [file cjasn-18-435-s001.pdf]
